# Surgical Management of Failed Roux-en-Y Gastric Bypass (RYGB) Reversal: A Case Study

**DOI:** 10.7759/cureus.36477

**Published:** 2023-03-21

**Authors:** Angie S Kim, Matthew Nester, Kristina T Gemayel, Joseph A Sujka

**Affiliations:** 1 Medicine, University of South Florida Morsani College of Medicine, Tampa, USA; 2 Plastic Surgery, University of South Florida/Tampa General Hospital, Tampa, USA; 3 General, Bariatric, Foregut, Hernia, University of South Florida/Tampa General Hospital, Tampa, USA

**Keywords:** gastrogastric stenosis, chronic marginal ulcers, complication, bypass reversal, bariatric surgery, obesity

## Abstract

With the growing obesity epidemic, surgeons are performing more bariatric surgeries, including Roux-en-Y gastric bypass (RYGB) reversals. Although studies have identified indications for RYGB reversals, little information is available about the long-term effects of the procedure. We wish to highlight a case with long-term complications of RYGB reversal and subsequent management.

We present a patient with multiple abdominal surgeries including an RYGB reversal that was complicated by a stenosed gastrogastric anastomosis that caused several gastrojejunostomy ulcerations and malnutrition secondary to intractable nausea and vomiting.

A 51-year-old female with a complex surgical history including a simple RYGB reversal in 2019 presented to the ER with complaints of abdominal pain, uncontrolled diarrhea, and an inability to tolerate food for six months. Workup revealed multiple marginal ulcers at the remnant jejunum attached to the gastric pouch, and a stenosed gastrogastrostomy placed high along the cardia of the remnant stomach and pouch. This stenosis resulted in a nonfunctional, nondependent reversal that only drained when filled. Ultimately, a large gastrotomy was performed, and an endoscope was utilized to identify a small pinhole connection between the patient’s pouch and the remnant stomach along the superomedial portion of the remnant stomach’s fundus. The anvil of a 60 mm GIA black load stapler was guided through and fired twice to come across the stricture. After the stricture was completely crossed, the endoscope was passed through, confirming that it was widely patent. The postoperative course was uneventful, and the patient was discharged with total parenteral nutrition (TPN) on postoperative day 15 before being discontinued at her follow-up visit. She reported that she had been gaining weight and eating well.

Long-term complications following RYGB reversal are not well-discussed in the literature. This case offers insight into such complications, discusses the surgical technique utilized to fix them, and calls for further research on the topic to better inform surgeons and patients alike.

## Introduction

Obesity is an epidemic that continues to affect medical treatment in all areas of healthcare. Bariatric surgery, in particular Roux-en-Y gastric bypass (RYGB), is one area that has experienced dramatic growth in the last decade, driven by not only the surging number of surgical candidates, but also because of its safety, low morbidity, and low mortality. While RYGB is a fairly safe and effective method for treating obesity, there have been instances in which patients decide to undergo reoperations for various reasons including weight recurrence or other complications [1]. It is estimated that up to 25% of bariatric patients will require a second operation at some point including reversal operations [2]. RYGB reversal is a second operation that bariatric patients may receive to resolve complications of RYGB including gastrojejunostomy anastomotic stricture, marginal ulcerations, gastrogastric fistula, nutritional deficiencies, and weight gain [3]. The procedure often involves reverting a patient’s anatomy to normal anatomy by removing the gastrojejunostomy and adding a new anastomosis between the gastric pouch and the remnant stomach.

Despite the growing number of RYGB reversals being performed in response to persistent complications, there is limited information available and thus no standardized guidelines for determining whether RYGB reversal is appropriate for patients. The decision to reverse rather than revise or convert is highly individualized, and it is unclear whether this is patient-driven, surgeon-driven, or a combination of both [1]. Furthermore, most studies regarding RYGB reversal highlight the rationale behind the operation rather than the outcomes, making it even more difficult to decide on re-operative surgery. This is especially true of patients who have had multiple abdominal surgeries, or at the very least an initial RYGB and subsequent revisional surgery, because they are more likely to experience abdominal adhesions as well as intra- and/or post-operative complications. For these patients, careful consideration prior to surgery is necessary. Consequently, there is a need for a better understanding of complications after reversing an RYGB and the possible interventions for them to better guide patients on their decision on whether a reversal (rather than a revision, for instance) is appropriate for them.

The following case study illustrates the complications of post-RYGB reversal leading to additional surgical revisions. Although this case does not fully encompass all the problems of a post-RYGB reversal, it does reveal the occurrence of one such problem in addition to the process of patient care and problem resolution as approached by bariatric surgeons.

This article will be presented as a poster presentation at the 2023 SAGES Annual Meeting on March 29, 2023.

## Case presentation

A 51-year-old female presented to the emergency department with complaints of abdominal pain, chronic uncontrolled diarrhea, and an inability to tolerate food for the past six months. During those six months, she became TPN dependent and was taking pantoprazole. Upon physical examination, her abdomen was soft with tenderness in the epigastric area. The patient’s surgical history was complex, consisting of an RYGB in 2003 (BMI: 48.9 kg/m^2^) and partial colectomy with an ostomy for colon cancer which was complicated by multiple re-do procedures. In 2019, she had several revisional operations that included an RYGB reversal to normal anatomy without modification to sleeve gastrectomy (BMI: 18.5 kg/m^2^), ileostomy reversal, ileorectal anastomotic stricture status post-dilation and stent placement. Her most recent operation prior to the presentation was an abdominoplasty in March 2022.

Initial work-up (BMI: 29.0 kg/m^2^) included a CT scan of the abdomen with contrast that revealed extensive postsurgical changes in the abdomen with diffuse small bowel dilation and narrowing at the ileorectal anastomosis. Initial esophagogastroduodenoscopy (EGD) was performed by gastroenterology which demonstrated a large anastomotic ulceration at the remnant jejunum of the gastric pouch and a patent, but difficult to access, reversal into the remnant stomach. In addition, a sigmoidoscopy was performed to rule out a distal stricture due to the patient’s complaint of diarrhea. No obstruction was evidenced, and it was suspected that the patient’s chronic diarrhea was functional in nature given her reconstructions.

**Figure 1:**
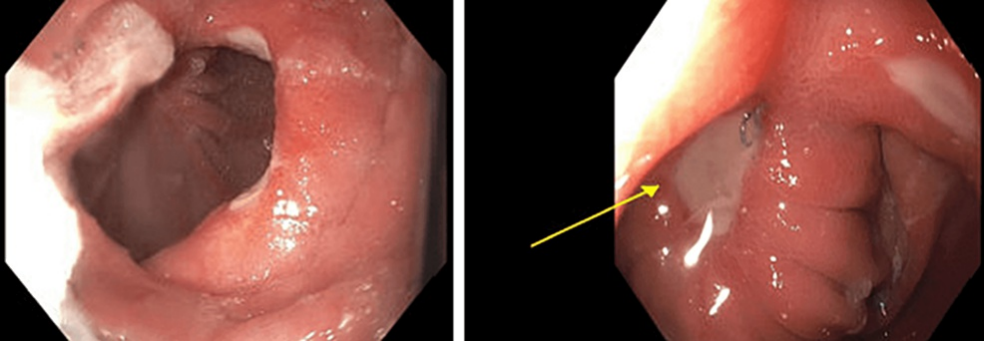
Multiple marginal ulcers are found at the patient's remnant jejunum at the gastric pouch upon upper endoscopy.

**Figure 2:**
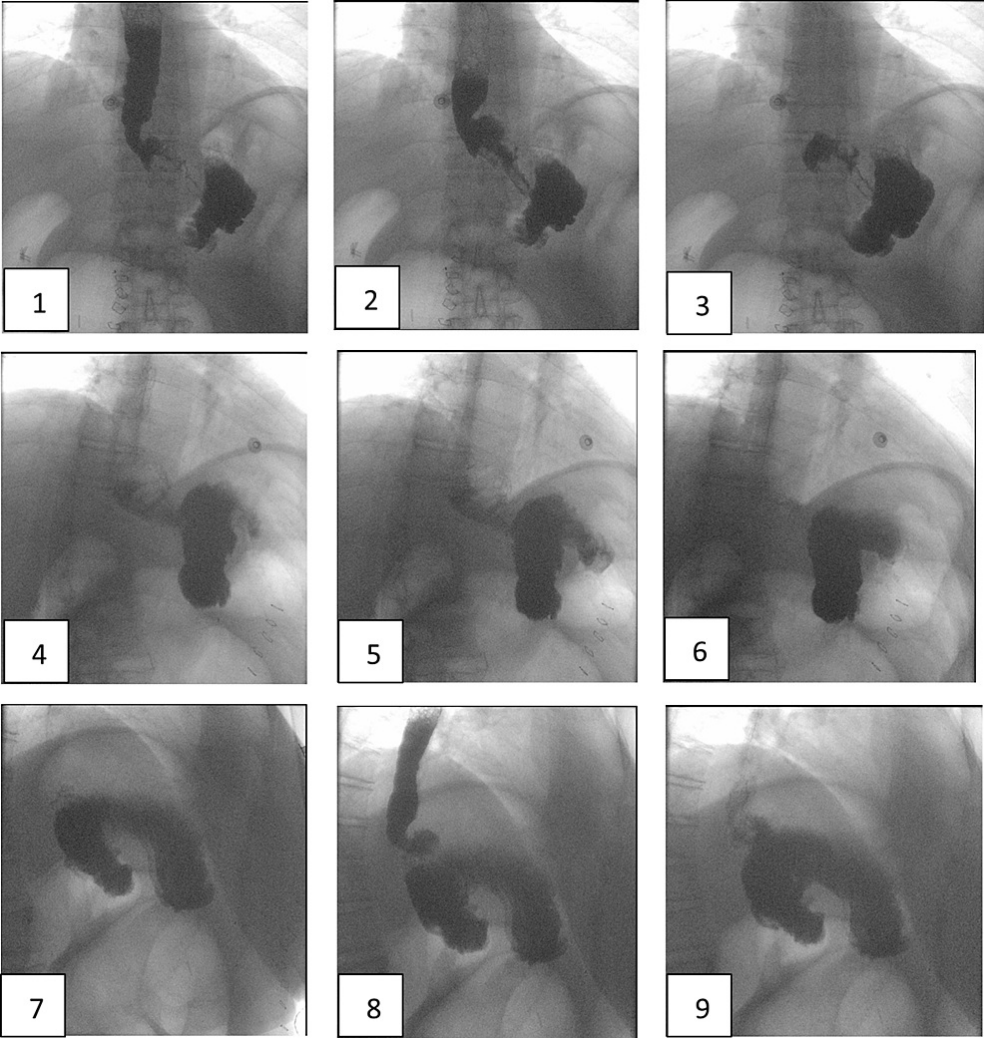
Upper gastrointestinal fluoroscopy series showing patient’s gastric pouch that drains into remnant stomach only after it is filled.

**Figure 3:**
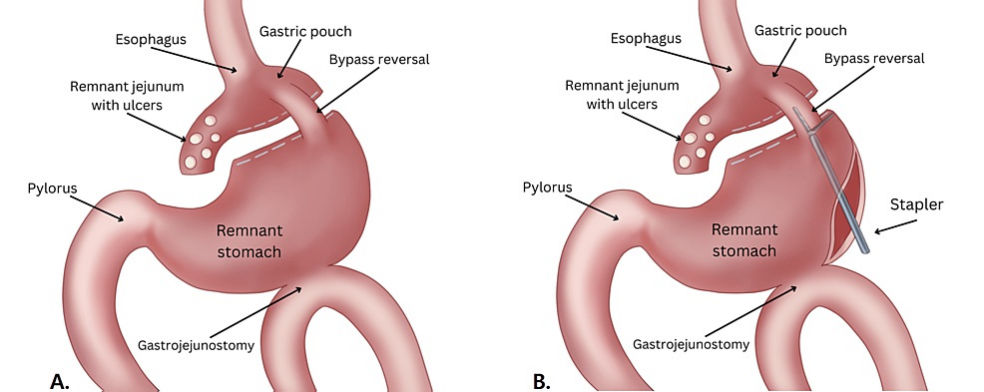
Original anatomical illustration by Rena Jiang and Kristina Gemayel of the patient’s upper gastrointestinal system. (A). Patient's anatomy after her RYGB reversal in 2019, revealing marginal ulcers at the remnant jejunum of the gastric pouch and a nonfunctional RYGB reversal. (B) For patient's RYGB reversal revision as detailed in this paper, a large gastrotomy was performed to find and widen the stenosed bypass reversal with a stapler.

Due to the patient’s ongoing symptoms, a repeat endoscopy was performed by surgery which showed ulcers at the patient’s remnant jejunum at the gastric pouch (Figure 1). Additionally, the gastrogastrostomy was not patent with only a small cuff of small bowel anastomosed to the pouch. Attempts to access the remnant stomach through the gastrogastrostomy were unsuccessful. Post-procedure, an upper gastrointestinal series was performed to evaluate the functionality of the patient’s gastrogastrostomy (Figure 2). This revealed that the previous gastric pouch needed to be filled completely before overflowing into the patient’s remnant stomach through her reversal. The patient’s gastrogastrostomy was placed high along the cardia of the remnant stomach and pouch and was therefore not dependent or particularly functional (Figure 3A). Based on these findings, the patient’s gastrojejunostomy ulceration was thought to be due to stasis in the gastric pouch, whereas her nausea and vomiting were due to an inability of her gastric pouch to drain easily into the remnant stomach.

Given these findings and her complex history, we discussed at length with the patient about operative intervention that included attempting to open the stenosis of her gastrogastrostomy RYGB reversal to improve drainage of her pouch. Upon surgery, the left upper quadrant of the abdomen was initially explored laparoscopically. However, ongoing attempts to mobilize the abdomen with the laparoscope were only partially successful and the decision was made to convert to an open procedure. The abdomen was entered through the patient’s previous laparotomy incision. Due to severe adhesive disease, dissection was difficult. Once it appeared that the remnant stomach had been identified, a laparoscopic port was placed into the remnant stomach, and it was insufflated. Examination revealed the patient’s anatomy, allowing for visualization of the patient’s closed gastrojejunostomy, pylorus, and duodenum. However, her gastric pouch, remnant jejunum, and gastrogastrostomy remained unidentified. Because the patient’s connection between the remnant stomach and gastric pouch was unable to be identified, a large gastrotomy was performed on the posterior aspect of the patient’s remnant stomach. An endoscope was utilized to discover a small pinhole connection between the patient's pouch and remnant stomach along the superomedial portion of the remnant stomach’s fundus at approximately 11 o’clock direction. With this direct visualization, a silk suture was tied around the endoscope and pulled through to the area of concern. The remnant jejunum of the gastric pouch continued to be difficult to visualize and was not resected at this time.

The anvil of a 60-mm GIA black load stapler was passed through the stricture, following the endoscope as a guide. Two fires of the 60mm GIA black load stapler were utilized to come across the stricture (Figure 3B). After the stricture was completely crossed, the endoscope was passed through this area to confirm that it was widely patent. The gastrotomy was then closed with multiple fires of 60 mm GIA purple loads. A leak test was performed, and no leak was identified. Once the stomach was closed, the endoscope was passed again through this area of stricture and verified that it was much wider than before. The patient was then closed and taken to recovery. Blood loss of this operation was approximately 100 cc and the operative time was four hours.

The patient’s hospital course was complicated by persistent abdominal pain and episodes of emesis and nausea, either due to residual ulcers at the remnant jejunum of the gastric pouch or her recent ileus complication. She was discharged home on post-operative day 15 (June 4, 2022) with TPN. Although the patient returned to the hospital with complaints of abdominal pain and wound dehiscence, this was resolved soon after wound drainage and incision before being discharged. At her follow-up visit on June 27, 2022, the patient’s TPN was discontinued. She has been gaining weight and eating well ever since.

## Discussion

With the increase in RYGB procedures each year due to the ongoing obesity epidemic, it is inevitable that patients will experience failures or complications of the operation and, eventually, a second operation. Often, re-operative surgery consists of a reversal, revision, or conversion of the problematic bariatric operation. The decision to reverse, rather than revise or convert, has been steadily growing and is motivated by the perspective that revisions would also be problematic or persistent [1, 4-6]. However, there are no standardized guidelines for deciding on an RYGB reversal. Decisions are largely based on indications for a reversal, which include the presence of marginal ulcers, malnutrition, anatomic complications, and functional complications such as chronic unexplained pain [1-3,6-14], as well as the outcomes of the procedure. It is evident that once a reversal to normal anatomy is made, symptoms do resolve for some, but not all, patients [2,15]. However, whether this absence of complications was maintained needs to be taken into consideration. Currently, there is a paucity of information available on late complications (> 30 days, post-operatively) following RYGB reversal.

The lack of data on outcomes of RYGB reversal to normal anatomy appears to be mostly attributed to the limited follow-up of the procedure, ranging from none to 41 months [15]. At present, most studies have discussed early complications (< 30 days, post-operatively), which include gastrogastric anastomotic leak, sepsis, and bleeding. The largest, single institute study to date reported a significant early complication rate of 29% [16]. Very few studies noted late complications, whereas those that did mostly commented on the resolution of the patient’s preoperative complications which initially led to the reversal [15,17]. Vilallonga et al. reported the incidence of other long-term complications, citing a new-onset of gastro-esophageal reflux in three out of 20 (15%) patients and chronic diarrhea in one out of 20 (5%), closely resembling the post-RYGB reversal complications of the patient in this case study. Furthermore, the challenging nature of the procedure itself can lend to other perioperative complications, due to limited field of vision because of multiple intestinal adhesions and the abnormal anatomy that many of these patients present with, which may subsequently transform into long-term complications. Overall, it is apparent that reversal is fraught with an elevated risk for postoperative complications [2,18]. The case report presented here, therefore, provides a longer-than-normal glimpse into complications following RYGB reversal, as the patient presented to the hospital two and a half years after her reversal.

The patient in this case study presented with pre-RYGB reversal complications of recurrent anastomotic ulceration. At present, the pathogenesis of these recurrent marginal ulcers remains unclear and hypotheses range from mechanical causes (pouch size, surgical technique) to ischemia and the presence of the foreign body at the anastomosis (such as staples, sutures, or Helicobacter pylori infection post-surgery) [3,7,10]. Because the patient’s recalcitrant ulcers were not resolved through medical therapy, she had clear indications for an RYGB reversal, as delineated in multiple studies [1-2,12,16]. Unfortunately, her pre-operative symptoms did not improve after the reversal with an additional post-reversal complication finding of a stenosed gastrogastric anastomosis and malnutrition secondary to intractable nausea and vomiting that required her to be placed on TPN.

These findings display a clear pathophysiological mechanism to the patient’s presentation. With the reversal being stenotic, the contents of the gastric pouch are unable to drain easily into the rest of the stomach, thereby resulting in severe gastroesophageal reflux. The stasis of fluid in the gastric pouch also gave rise to the gastrojejunostomy ulcer or marginal ulcer. It is interesting to note that few, if any cases, cite stenosis of the gastrogastric anastomosis as a post-operative complication requiring surgery. Although it is possible that the stenosis occurred due to an external pressure (i.e., lymph node enlargement) due to her history of colon cancer, this is unlikely since she presented to clinic 15-20 years after her initial diagnosis and there was no evidence of cancer recurrence on her numerous CT scans. Furthermore, the complication does not seem to be outside the realm of possibility since there have been cases of severe gastrojejunal anastomosis strictures due to marginal ulcers after RYGB procedures which, after multiple rounds of conservative treatment, eventually required a RYGB reversal [6]. Likewise, in this case, a conservative or less invasive procedure could have been attempted prior to converting to an open procedure. Additional endoscopic interventions could have been done to mobilize the gastrogastric anastomosis or possibly an endoscopic ultrasound-directed transgastric ERCP (EDGE) procedure. Otherwise, an open procedure may have been the safest option due to the complexity of this patient’s revision of RYGB reversal due to the complex changes in anatomy, such as the inaccessibility of the patient’s remnant stomach. Furthermore, the multiple adhesions that exist from prior surgical interventions would have made it difficult for a less invasive procedure.

## Conclusions

It is remarkable that there is so little information on the complications of post-RYGB reversal, especially when there appears to be a variety of complications associated with post-RYGB that eventually results in the decision to reverse. This requires multiple surgeries followed by risks for several post-operative complications that may place a heavy burden on the patient. Many studies have shown that reversal to normal anatomy after RYGB is safe and effective. However, there is a need for longer follow-ups to confirm such findings with a higher level of evidence and to address the possibility of other late post-RYGB reversal complications to better prepare physicians for the care of patients like the patient in this case.
